# Lung Thermal Ablation: Comparison between an Augmented Reality Computed Tomography (CT) 3D Navigation System (SIRIO) and Standard CT-Guided Technique

**DOI:** 10.3390/biology10070646

**Published:** 2021-07-11

**Authors:** Rosario Francesco Grasso, Flavio Andresciani, Carlo Altomare, Giuseppina Pacella, Gennaro Castiello, Massimiliano Carassiti, Carlo Cosimo Quattrocchi, Eliodoro Faiella, Bruno Beomonte Zobel

**Affiliations:** 1 Department of Diagnostic and Interventional Radiology, University Hospital Campus Bio-Medico of Rome, Via Alvaro del Portillo, 200, 00128 Rome, Italy; f.andresciani@unicampus.it (F.A.); c.altomare@unicampus.it (C.A.); g.pacella@unicampus.it (G.P.); g.castiello@unicampus.it (G.C.); c.quattrocchi@unicampus.it (C.C.Q.); e.faiella@unicampus.it (E.F.); b.zobel@unicampus.it (B.B.Z.); 2 Unit of Anesthesia, Intensive Care and Pain Management, University Hospital Campus Bio-Medico of Rome, Via Alvaro del Portillo, 200, 00128 Rome, Italy; m.carassiti@unicampus.it

**Keywords:** lung ablation, navigation system, microwave ablation, radiation dose, radiofrequency

## Abstract

**Simple Summary:**

Lung cancer is the leading cause of cancer mortality worldwide. In recent years, numerous technologies have been used to perform image-guided percutaneous thermal ablation, mainly including radiofrequency ablation, microwave ablation, and cryoablation. These image-guided ablation techniques have emerged as a safe, cost-effective, minimally invasive treatment alternative for patients who do not require surgery. Procedural planning, monitoring, and lesion targeting are generally performed with the help of computed tomography; navigation systems are emerging as valid tool to reduce procedural time and radiation dose administration. In the present paper, we investigate the efficacy of an optical-based navigation system (SIRIO) to perform lung thermal ablation. SIRIO proved to be a reliable and effective tool when performing CT-guided LTA, displaying a significant decrease in the number of required CT scans, procedure time, and radiation doses administered to patients.

**Abstract:**

(1) Background: The aim of this retrospective study is to assess safety and efficacy of lung radiofrequency (RFA) and microwave ablation (MWA) using an augmented reality computed tomography (CT) navigation system (SIRIO) and to compare it with the standard CT-guided technique. (2) Methods: Lung RFA and MWA were performed with an augmented reality CT 3D navigation system (SIRIO) in 52 patients. A comparison was then performed with a group of 49 patients undergoing the standard CT-guided technique. All the procedures were divided into four groups based on the lesion diameter (>2 cm or ≤2 cm), and procedural time, the number of CT scans, radiation dose administered, and complications rate were evaluated. Technical success was defined as the presence of a “ground glass” area completely covering the target lesion at the immediate post-procedural CT. (3) Results: Full technical success was achieved in all treated malignant lesions for all the considered groups. SIRIO-guided lung thermo-ablations (LTA) displayed a significant decrease in the number of CT scans, procedure time, and patients’ radiation exposure (*p* < 0.001). This also resulted in a dosage reduction in hypnotics and opioids administrated for sedation during LTA. No significant differences were observed between the SIRIO and non-SIRIO group in terms of complications incidence. (4) Conclusions: SIRIO is an efficient tool to perform CT-guided LTA, displaying a significant reduction (*p* < 0.001) in the number of required CT scans, procedure time, and patients’ radiation exposure.

## 1. Introduction

Over the past two decades there has been a progressive increase in the number of patients treated with percutaneous lung thermal ablation (LTA) [1,2,3,4,5,6]. This technique is applied in early-stage non-small cell lung carcinoma (NSCLC) for non-surgical patients, local recurrence, or oligometastatic patients [1,2,3,4,5,6,7,8,9,10,11,12,13]. The main technologies applied today for LTA are radiofrequency ablation (RFA), microwave ablation (MWA), and cryoablation (CA) [1,14,15,16,17,18].

Procedural planning, monitoring, and lesion targeting are generally performed with the help of computed tomography (CT) [1,14,19]. More recently, the implementation of C-arm cone-beam CT (CBCT) technology has introduced a new image guidance strategy, even though concerns are still raised due to the significant radiations amount to patients and operators [19].

Thus, navigation systems are emerging as a valid tool to reduce procedural times and administration of radiation doses, allowing electromagnetic [20,21], optical [22,23,24], or hybrid tracking [25,26,27] of the devices used during percutaneous interventions and their real-time visualization in a model obtained from a previously acquired CT scan.

In the present paper, we investigate the efficacy of an optical-based navigation system (SIRIO, MASMEC S.p.A., Modugno, BA, Italy, http://www.masmec.org/ accessed on 1 July 2021) to perform LTA.

To our knowledge, this is the first experience centered around CT-guided lung thermo-ablation performed with an optical navigation system in a large cohort of patients.

## 2. Materials and Methods

### 2.1. Populations

This study was accepted by our local institutional review board; all the enrolled patients provided their written informed consent to undergo lung thermo-ablation. Data regarding patients referred to our department for LTA due to primary or metastatic biopsy-proven lung tumors between January 2010 and November 2020 were retrospectively reviewed. Inclusion criteria were as follows: solid lesions or ground-glass opacities biopsy-proved to be malignant, unsuitable for surgical approach, and previously discussed in a tumor board.

SIRIO-guided LTA were performed in 52 patients (29 male, 22 female, mean age 66.8 ± 12 years) and data collected from this group were compared with those obtained from the cohort of 48 patients (27 male, 21 female, 69.1 ± 10 years), who received standard CT-guided LTA. For each group we also considered two subgroups based on the size of the lesions, according to a cut-off of 2 cm (>2 cm or ≤2 cm); extensive population characteristics are displayed in Table 1.

### 2.2. Procedure

The navigation system was installed in a CT suite (Somaton Sensation 64, Siemens, Forchheim, Germany). Parameters used were 64 × 0.6-mm detector configuration, pitch was 1.45, table speed was 2.54 mm/rotation, gantry rotation was 0.33-s, 100 kV and 100 mAs, with a CT scan restricted to the target area and a soft tissue kernel, predictable by the mean of the previous radiological records available for each single patient.

Two radiologists with more than 10 years of experience in percutaneous ablation performed LTA (R.F.G. and E.F.) under local anesthesia (10–20 mL mepivacaine hydrochloride 2% on the parietal surface of the pleura) and deep sedation. Dosage of hypnotics and opioids for sedation during LTA were as follows: 0.5–1 mg/kg of propofol was used as a loading dose, 1–3 mg/kg/h as maintenance; 0.02–0.1 mg/kg of midazolam was used as a bolus dose, and 0.5–1 mcg/kg of fentanyl was also used as a bolus dose, with 25–30 min of time interval. Conscious sedation was preferred to general anesthesia as the latter increases the procedure’s total cost, duration, and pneumothorax risk when positive pressure is used [28,29]. Moreover, conscious sedation performed after the probe positioning ensures a better control over patient’s respiratory motion, as the radiologist can ask the patient not to breathe while positioning the probe.

#### 2.2.1. Standard CT-Guided Procedures 

A row of needles were positioned on the patient’s chest to mark the sagittal plane and the CT gantry laser line was used to achieve spatial orientation. When the path was chosen, the physician advanced the needle in small steps, with a reimaging performed to measure the new needle location after each advancement.

#### 2.2.2. SIRIO-Guided Procedures

**Figure 1:**
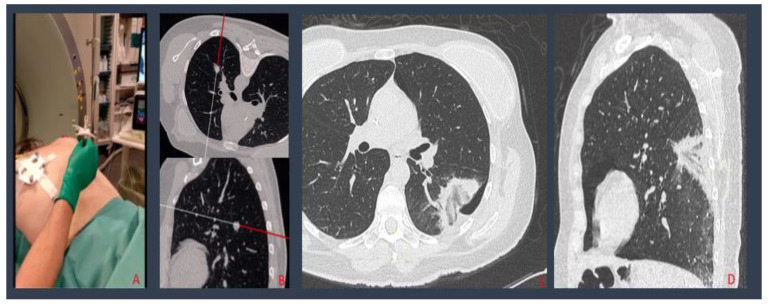
Percutaneous lung MW ablation of a breast cancer metastasis: needle and patient tools positioning (**A**); the navigation system, creating a 3D virtual model of the patient’s chest, where needle progressions into the patient’s chest are shown (**B**); post procedural MPR CT reconstructions displaying a small PNX adjacent to the ablation zone, without evidence of residual disease (**C**,**D**).

A photo sensor fixed on the CT room ceiling which perceives infrared light reflected by passive spheres was placed on the needle handgrip and on the patient’s chest (Figure 1); this ensured that needle progressions towards the lesion were recognized and displayed on a 3D virtual model of the patient’s chest basing on DICOM CT images reconstructed by the navigation system. This resulted in needle advancement without the need for a new CT acquisition for each movement.

A last CT scan was acquired at the end of all the procedures to assess technical success of the procedure and complications, e.g., pneumothorax (PNX), hemoptysis, pulmonary hemorrhage, hemothorax, and pleural effusion. If the patient developed no symptoms after the ablation, a chest CT scan was performed 12–24 h following the LTA and the patient was dismissed.

### 2.3. Data Collection

The patients’ age and sex, and procedure-related data such as lesion size and location, were collected (Table 1). The number of CT scans for each procedure was obtained by the data saved on the local picture archiving and communication system (PACS). Radiation exposure was estimated by the total dose-length product (TDLP) obtained by summing the DLP of each CT scan performed during the entire PLB procedure; then, the following formula was used to obtain the dose:

Effective Radiation Dose = TDLP * conversion factor k (chest; 0.017 mSv * mGy^−1^ * cm^−1^) [22].

Procedural time was assessed by recording the difference between the clock reading on the scout view and the time on the last acquired CT-scan performed after the removal of the ablation instrument.

Technical success was defined as the presence of a “ground glass” area completely covering the target lesion at the immediate post-procedural CT. This imaging appearance was a rough approximation of margin of tissue necrosis, thus suggesting if the lesion was treated with a safety margin of approximately 1 cm [1,30,31].

Dosage of hypnotics and opioids administer for sedation during each procedure were recorded. Data regarding major complications (PNX or massive pleural effusion which required drain tube positioning, hemorrhages and broncho-pleural fistulas) were collected.

### 2.4. Statistics

The differences in terms of the number of CT scans, patient radiation exposure, and procedural time were analyzed between the procedures performed with SIRIO and without SIRIO in both dimensional groups (<2 cm and > 2 cm). A comparison between SIRIO and non-SIRIO groups was performed for each variable with the *t*-test (*p* < 0.01) and was represented by boxplot graphs.

All the statistics were elaborated using IBM SPSS Statistics for Windows, version 26 (IBM Corp., Armonk, NY, USA).

## 3. Results

We analyzed a total of 101 procedures; 52 were performed with a CT navigation system (SIRIO) and 49 with standard CT-guided technique.

Full technical success was achieved in all treated malignant lesions for all the considered groups.

A statistically significant reduction (*p* < 0.05) in the number of CT-scans, procedural time, and radiation dose was observed for LTAs performed under SIRIO guidance compared to those performed under standard CT-guidance, in both ≤2 cm and >2cm groups. Mean values of each variable and for each group are showed in Table 2. For lesions ≤2 cm we report the mean procedural time of 57.2 ± 9 min, the mean number of CT scans of 9.4 ± 2.7, and mean radiation dose of 14.8 ± 4.5 mSv for the non-SIRIO group, while, for SIRIO group, we report32.6 ± 11.8 min, 4.9 ± 2.3 CT scans, and 8 ± 4.5 mSv, respectively.

**Figure 2:**
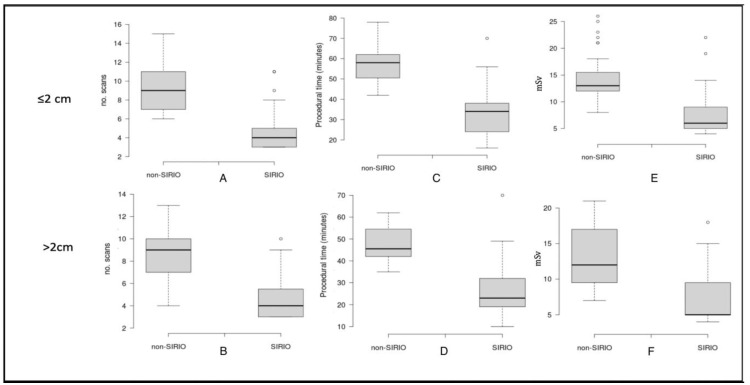
Boxplots showing comparison between mean number of CT-scans (**A**,**B**), procedural time (**C**,**D**) and radiation dose (**E**,**F**) for each group.

On the other hand, for lesions >2 cm we report the mean procedural time of 47.3 ± 8 min, the mean number of CT scans of 8.5 ± 2.2, and a mean radiation dose of 13.3 ± 4.3 mSv for the non-SIRIO group, while, for the SIRIO group, we report 27.2 ± 13.8 min, 4.7 ± 2 CT scans, and 7 ± 4 mSv, respectively. Boxplots of the three variables for each group are shown in Figure 2.

The decrease in procedural time in the SIRIO group determined a reduction in the required dose of hypnotics and opioids of about one-third of that used for the conventional CT-guided technique.

**Figure 3:**
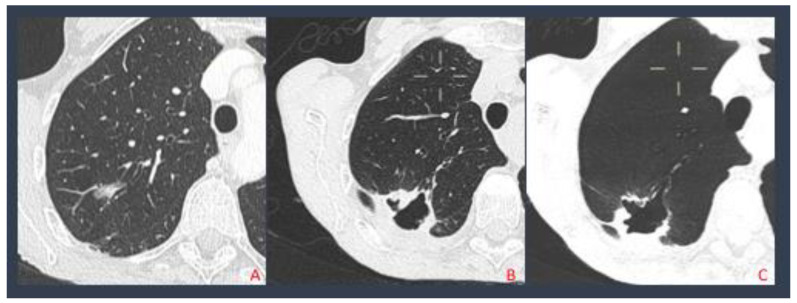
Pulmonary adenocarcinoma treated with MWA (**A**); after 4 months the patient developed a broncho-pleural fistula (**B**); MinPR reconstruction displaying the fistula (**C**).

Complication types and the relatives’ rate are shown in Table 3. We report no complications in the non-Sirio group in 43 cases, 12 PNX cases, 4 cases of pleural effusions, and 1 case of hemopericarium and broncho-pleural fistula. In the SIRIO group, we report 30 cases without complications, 6 cases of PNX, and 1 case of pleural effusion, massive subcutaneous emphysema, and broncho-pleural fistula (Figure 3).

## 4. Discussion

CT is the most frequent technique employed to guide LTA which is still raising concerns about the administration of a radiation dose to patients and the lack of real-time control during the positioning of ablation probes. Fluoroscopic guidance could represent a solution to track the instrumentation movements in the patient; however, it would not solve the issue of patient irradiation and would add radiation exposure also for the operating personnel [1,2,9].

In recent years, several guidance systems based on augmented reality navigation have been introduced in different surgical settings [20,21,22,23,24,25,26,27], including SIRIO, which has already been validated as an effective tool for the guidance of biopsy procedures on lung lesions [22,24]. To our knowledge, this is the first study in which SIRIO is evaluated as a tool to assist percutaneous LTA in comparison to standard CT-guidance which represents the most used technique.

Our results show a significant reduction in the number of CT scans, procedural time, and radiation dose in procedures performed under SIRIO guidance, permitting to reduce the number of intra-procedural CT acquisitions to assess the instrument’s position, thus reducing overall procedural times and radiation doses for the patient.

Moreover, our data display that the reduction in all variables for SIRIO-guided procedures is greater for lesions ≤2 cm, as showed by boxplot graphs and p values calculated applying statistical tests (*t*-test). This suggests the efficacy of SIRIO guide in reaching smaller lesions; standard CT-guidance, on the contrary, is less efficient for small lesions for which a high number of intra-procedural CT scans are required. Complications incidence did not significantly differ for lesions >2 cm (*p* > 0.05), even though SIRIO guides ensured 87.5% of LTAs without complications for lesions ≤2 cm (vs. 68.8%), demonstrating that a real-time control during ablative procedures guarantee a lower complications incidence.

Furthermore, the considerable reduction in time resulted in a reduction in the maintenance dose of hypnotics and opioids administrated by about one-third of that used for the conventional CT-guided technique. This allows for improvements in interventional room occupancy and medical and paramedical staff employment, resulting in significant cost savings for the hospital.

This study has some limitations: SIRIO-guided procedures were retrospectively evaluated as the standard CT-guided procedure were performed before the availability of SIRIO at our department. Moreover, procedural time for LTA includes the ablation time after the instrument positioning within the lesions, which may differ between RFA and MWA. However, the numbers of procedures performed using the different techniques are similar between the SIRIO and non-SIRIO groups in both dimensional classes. Other limitations include the absence of multi-center involvement and randomization. Other than for lungs, SIRIO use may be considered to guide procedures on other parenchymatous organs and to reach deep anatomical compartments.

## 5. Conclusions

SIRIO proved to be a reliable and effective tool when performing CT-guided LTA, displaying a significant (*p* < 0.001) decrease in number of required CT scans, procedure time, and radiation dose administered to patients. Our future perspective is to demonstrate that navigation tools also improve clinical outcomes in terms of local tumor control.

## Figures and Tables

**Table 1:** Displaying the mean maximum diameter, histology and localization of lesions for each group (RUL = right upper lobe, ML = medial lobe, RLL = right lower lobe, LUL = left upper lobe, LLL = left lower lobe).

				Lobe	N Cases	%
CT < 2 cm (*n* = 32)	lesion diameter (cm)	mean	SD	RUL	8	25
		13.75	3.86047	ML	3	9.4
	Histology	N cases	%	RLL	6	18.8
	metastases	27	84.4	LUL	5	15.6
	adenocarcinoma	3	9.4	LINGULA	1	3.1
	squamous carcinoma	2	6.3	LLL	9	28.1
CT > 2 cm (*n* = 29)	lesion diameter (cm)	mean	SD	RUL	4	25
		34.94	10.50377	ML	0	0
	Histology	N cases	%	RLL	4	25
	metastases	8	50	LUL	2	12.5
	adenocarcinoma	5	31.3	LINGULA	1	6.3
	squamous carcinoma	3	18.8	LLL	5	31.3
SIRIO < 2 cm (*n* = 16)	lesion diameter	mean	SD	RUL	8	27.6
		13.38	4.32	ML	2	6.9
	Histology	N cases	%	RLL	9	31
	metastases	16	55.2	LUL	8	27.6
	adenocarcinoma	8	27.6	LINGULA	0	0
	squamous carcinoma	5	17.2	LLL	2	6.9
SIRIO > 2 cm (*n* = 23)	lesion diameter (cm)	mean	SD	RUL	7	30.4
		35.74	13.47461	ML	3	13
	Histology	N cases	%	RLL	1	4.3
	metastases	11	47.8	LUL	5	21.7
	adenocarcinoma	6	26.1	LINGULA	1	4.3
	squamous carcinoma	6	26.1	LLL	6	26.1

**Table 2:** Showing mean procedural time, number of CT scans and Radiation Dose for each group.

Lesions ≤ 2 cm					
Variable	Non-SIRIO		SIRIO		*p* value
	Mean	SD	Mean	SD	
Time (min)	57.25	9	32.65	11.8	<0.005
No. scans	9.47	2.78	4.93	2.37	<0.005
Dose (mSv)	14.87	4.50	8	4.59	<0.005
**Lesions > 2 cm**					
Variable	Non-SIRIO		SIRIO		*p* value
	Mean	SD	Mean	SD	
Time (min)	47.375	8.421203398	27.2608696	13.45773651	<0.005
No. scans	8.50	2.25	4.74	2.03	<0.005
Dose (mSv)	13.36	4.38	7	4.02	<0.005

**Table 3:** Complication types and relative rates.

	Complication	N Cases	%
CT < 2 cm (*n* = 32)	None	22	68.8
	PNX	5	15.6
	Pleural effusion	3	9.4
	Hemopericardium	1	3.1
	Broncho-pleural fistula	1	3.1
CT > 2 cm (*n* = 29)	None	21	72.4
	PNX	7	24.4
	Pleural effusion	1	3.2
	Hemopericardium	0	0
	Broncho-pleural fistula	0	0
SIRIO < 2 cm (*n* = 16)	None	14	87.5
	PNX	2	12.5
	Pleural effusion	0	0
	Hemopericardium	0	0
	Broncho-pleural fistula	0	0
SIRIO > 2 cm (*n* = 23)	None	16	69.9
	PNX	4	17.2
	Pleural effusion	1	4.3
	Massive subcutaneous emphysema	1	4.3
	Broncho-pleural fistula	1	4.3

## Data Availability

The data presented in this study are available on request from the corresponding author. The data are not publicly available due to privacy restrictions.

## References

[1] Venturini M., Maurizio C., Marra P., Salvatore M., Pereira P.L., Gianpaolo C. (2020). CIRSE Standards of Practice on Thermal Ablation of Primary and Secondary Lung Tumours. Cardiovasc. Interv. Radiol..

[2] Páez-Carpio A., Gómez F.M., Olivé G.I., Paredes P., Baetens T., Carrero E., Sánchez M., Vollmer I. (2021). Image-guided percutaneous ablation for the treatment of lung malignancies: Current state of the art. Insights Imaging.

[3] Postmus P.E., Kerr K.M., Oudkerk M., Senan S., Waller D.A., Vansteenkiste J., Escriu C., Peters S. (2017). Early and locally advanced non-small-cell lung cancer (NSCLC): ESMO Clinical Practice Guidelines for diagnosis, treatment and follow-up. Ann. Oncol..

[4] Van Cutsem E., Cervantes A., Adam R., Sobrero A., Van Krieken J.H., Aderka D. (2016). ESMO consensus guidelines for the man-agement of patients with metastatic colorectal cancer. Ann. Oncol..

[5] Rosen J.E., Keshava H.B., Yao X., Kim A.W., Detterbeck F.C., Boffa D.J. (2016). The Natural History of Operable Non-Small Cell Lung Cancer in the National Cancer Database. Ann. Thorac. Surg..

[6] Louie A.V., Palma D.A., Dahele M., Rodrigues G.B., Senan S. (2015). Management of early-stage non-small cell lung cancer using stereotactic ablative radiotherapy: Controversies, insights, and changing horizons. Radiother. Oncol..

[7] Engelhardt K.E., Feinglass J.M., DeCamp M.M., Bilimoria K.Y., Odell D.D. (2018). Treatment trends in early-stage lung cancer in the United States, 2004 to 2013: A time-trend analysis of the National Cancer Data Base. J. Thorac. Cardiovasc. Surg..

[8] Haasbeek C.J.A., Palma D., Visser O., Lagerwaard F.J., Slotman B., Senan S. (2012). Early-stage lung cancer in elderly patients: A population-based study of changes in treatment patterns and survival in the Netherlands. Ann. Oncol..

[9] Palussière J., Chomy F., Savina M., Deschamps F., Gaubert J.Y., Renault A., Bonnefoy O., Laurent F., Meunier C., Bellera C. (2018). Radiofrequency ablation of stage IA non–small cell lung cancer in patients ineligible for surgery: Results of a prospective multicenter phase II trial. J. Cardiothorac. Surg..

[10] Dupuy D.E., Fernando H.C., Hillman S., Ng T., Tan A.D., Sharma A. (2015). Radiofrequency ablation of stage IA non-small cell lung cancer in medically inoperable patients: Results from the American College of Surgeons Oncology Group Z4033 (Al liance) trial. Cancer.

[11] Donington J., Ferguson M., Mazzone P., Handy J., Schuchert M., Fernando H. (2012). American College of Chest Physicians and Society of Thoracic Surgeons consensus statement for evaluation and management for high-risk patients with stage I non- small cell lung cancer. Chest.

[12] Sandler K.A., Abtin F., Suh R., Cook R.R., Felix C., Lee J.M., Garon E.B., Wu J., Luterstein E.M., Agazaryan N. (2018). A Prospective Phase 2 Study Evaluating Safety and Efficacy of Combining Stereotactic Body Radiation Therapy With Heat-based Ablation for Centrally Located Lung Tumors. Int. J. Radiat. Oncol..

[13] Howington J.A., Blum M.G., Chang A., Balekian A.A., Murthy S.C. (2013). Treatment of Stage I and II Non-small Cell Lung Cancer. Chest.

[14] Roy A.M., Bent C., Fotheringham T. (2009). Radiofrequency ablation of lung lesions: Practical applications and tips. Curr. Probl. Diagn. Radiol..

[15] Vogl T.J., Naguib N.N., Gruber-Rouh T., Koitka K., Lehnert T., Nour-Eldin N.E.A. (2011). Microwave ablation therapy: Clinical utility in treatment of pulmonary metastases. Radiology.

[16] Hinshaw J.L., Lubner M.G., Ziemlewicz T., Lee F.T., Brace C. (2014). Percutaneous Tumor Ablation Tools: Microwave, Radiofrequency, or Cryoablation—What Should You Use and Why?. Radiology.

[17] Palussière J., Catena V., Buy X. (2017). Percutaneous thermal ablation of lung tumors—radiofrequency, microwave and cryotherapy: Where are we going?. Diagn. Interv. Imaging.

[18] Macchi M., Belfiore M.P., Floridi C., Serra N., Belfiore G., Carmignani L. (2017). Radiofrequency versus microwave ablation for 87. treatment of the lung tumours: LUMIRA (lung microwave radiofrequency) randomized trial. Med Oncol..

[19] Cazzato R.L., Battistuzzi J.-B., Catena V., Grasso R.F., Zobel B.B., Schena E., Buy X., Palussiere J. (2015). Cone-Beam Computed Tomography (CBCT) Versus CT in Lung Ablation Procedure: Which is Faster?. Cardiovasc. Interv. Radiol..

[20] Wood B.J., Zhang H., Durrani A., Glossop N., Ranjan S., Lindisch D., Levy E., Banovac F., Borgert J., Krueger S. (2005). Navigation with Electromagnetic Tracking for Interventional Radiology Procedures: A Feasibility Study. J. Vasc. Interv. Radiol..

[21] Appelbaum L., Sosna J., Nissenbaum Y., Benshtein A., Goldberg S.N. (2011). Electromagnetic Navigation System for CT-Guided Biopsy of Small Lesions. Am. J. Roentgenol..

[22] Grasso R.F., Faiella E., Luppi G., Schena E., Giurazza F., Del Vescovo R., D’Agostino F., Cazzato R.L., Zobel B.B. (2013). Percutaneous lung biopsy: Comparison between an augmented reality CT navigation system and standard CT-guided technique. Int. J. Comput. Assist. Radiol. Surg..

[23] Faiella E., Castiello G., Bernetti C., Pacella G., Altomare C., Andresciani F., Zobel  B.B., Grasso R.F. (2021). Impact of an Augmented Reality Navigation System (SIRIO) on Bone Percutaneous Procedures: A Comparative Analysis with Standard CT-Guided Technique. Curr Oncol..

[24] Faiella E., Frauenfelder G., Santucci D., Luppi G., Schena E., Zobel B.B., Grasso R.F. (2018). Percutaneous low-dose CT-guided lung biopsy with an augmented reality navigation system: Validation of the technique on 496 suspected lesions. Clin. Imaging.

[25] Khan M.F., Dogan S., Maataoui A., Wesarg S., Gurung J., Ackermann H., Schiemann M., Wimmer-Greinecker G., Vogl T.J. (2006). Navigation-Based Needle Puncture of a Cadaver Using a Hybrid Tracking Navigational System. Investig. Radiol..

[26] Chehab M.A., Brinjikji W., Copelan A., Venkatesan A.M. (2015). Navigational Tools for Interventional Radiology and Interventional Oncology Applications. Seminars in Interventional Radiology.

[27] Meier-Meitinger M., Nagel M., Kalender W., Bautz W.A., Baum U. (2008). Computer-assisted navigation system for interventional CT-guided procedures: Results of phantom and clinical studies. Fortschritte auf dem Gebiete der Rontgenstrahlen und der Nuklearmedizin.

[28] Hiraki T., Gobara H., Fujiwara H., Ishii H., Tomita K., Uka M. (2013). Lung cancer ablation: Complications. Seminars in Interventional Radiology.

[29] Zheng A., Wang X., Yang X., Wang W., Huang G., Gai Y., Ye X. (2014). Major Complications after Lung Microwave Ablation: A Single-Center Experience on 204 Sessions. Ann. Thorac. Surg..

[30] Abtin F.G., Eradat J., Gutierrez A.J., Lee C., Fishbein M.C., Suh R.D. (2012). Radiofrequency Ablation of Lung Tumors: Imaging Features of the Postablation Zone. Radiology.

[31] Clasen S., Krober S.M., Kosan B., Aebert H., Fend F., Bomches A. (2008). Pathomorphologic evaluation of pulmonary radiofre- quency ablation. Cancer.

